# Wearing Compression Tights on the Thigh during Prolonged Running Attenuated Exercise-Induced Increase in Muscle Damage Marker in Blood

**DOI:** 10.3389/fphys.2017.00834

**Published:** 2017-10-26

**Authors:** Sahiro Mizuno, Mari Arai, Fumihiko Todoko, Eri Yamada, Kazushige Goto

**Affiliations:** ^1^Graduate School of Sports and Health Science, Ritsumeikan University, Shiga, Japan; ^2^Descente Ltd., Osaka, Japan; ^3^Faculty of Sports and Health Science, Ritsumeikan University, Shiga, Japan

**Keywords:** compression gear, prolonged running, exercise-induced muscle damage, jump performance, maximal muscular strength

## Abstract

**Purpose:** To examine the effects of wearing a lower-body compression garment with different body coverage areas during prolonged running on exercise performance and muscle damage.

**Methods:** Thirty male subjects were randomly assigned to one of three groups: (1) wearing a compression tights with 15 mmHg to thigh [*n* = 10, CT group], (2) wearing a compression socks with 15 mmHg to calf [*n* = 10, CS group], and (3) wearing a lower-body garment with < 5 mmHg to thigh and calf [*n* = 10, CON group]. The exercise consisted of 120 min of uphill running at 55% of $\dot{\text{V}}$O_2_max. Heart rate (HR), rate of perceived exertion (RPE), and running economy (evaluated by VO_2_) were monitored during exercise every 10 min. Changes in maximum voluntary contraction (MVC) of knee extension and plantar flexion, height of counter movement jump (CMJ) and drop jump (DJ), and scores of subjective feelings of muscle soreness and fatigue were evaluated before exercise, and 60 and 180 min after exercise. Blood samples were collected to determine blood glucose, lactate, serum free fatty acid, myoglobin (Mb), high-sensitivity C-reactive protein, and plasma interleukin-6 concentrations before exercise (after 20 min of rest), at 60 min of exercise, immediately after exercise, and 60 and 180 min after exercise.

**Results:** Changes in HR, RPE, and running economy during exercise did not differ significantly among the three groups. MVC of knee extension and plantar flexion, and DJ decreased significantly following exercise, with no difference among groups. The serum Mb concentration increased significantly with exercise in all groups, whereas the area under the curve for Mb concentration during 180 min post-exercise was significantly lower in the CT group (13,833 ± 1,397 pg/mL 180 min) than in the CON group (24,343 ± 3,370 pg/mL 180 min, *P* = 0.03).

**Conclusion:** Wearing compression garment on the thigh significantly attenuated the increase in serum Mb concentration after exercise, suggesting that exercise-induced muscle damage was attenuated.

## Introduction

The use of lower-body compression garments (e.g., full length, thigh length, and knee length) during running is becoming increasingly popular among athletes as a strategy to improve exercise performance. Accumulating evidence supports the benefits of wearing compression garments during exercise, including improved running economy, attenuated decrease in muscular power output, and enhanced removal of metabolites during running (Bringard et al., 2005; Ali et al., 2011; Miyamoto and Kawakami, 2014; Mizuno et al., 2017). Furthermore, recent publications have implicated that applied pressure plays a crucial role in the benefits of wearing compression garments (MacRae et al., 2011; Beliard et al., 2015). This notion is consistent with our recent findings (Mizuno et al., 2017), which revealed that wearing a compression garment that exerts 15 mmHg at the thigh and calf significantly attenuated the decrease in jump performance and prevented the increases in heart rate (HR) and interleukin-6 (IL-6) levels induced by 120 min of running. However, these effects were not observed when compression garments exerting 30 mmHg were applied. These results suggested that optimal compression intensity exists for improving exercise performance and attenuating the development of fatigue during prolonged exercise (Mizuno et al., 2017).

Increased peripheral circulation has been suggested as a plausible mechanism for attenuating exercise-induced fatigue, which can be accomplished by wearing compression garments during prolonged running (MacRae et al., 2012). External pressure by the garments assists muscle pump action, which enhances removal of intramuscular metabolites and venous return. Reduced muscle oscillation is another possible mechanism, because exercise-induced muscle oscillation may result in muscle fatigue and/or tissue damage (Kraemer et al., 1998; Doan et al., 2003). Accordingly, an increase in applied pressure at a distal site in the lower limb muscles is believed to facilitate peripheral blood circulation. Pressure applied to large working muscles would also be crucial for reducing muscle oscillation. In contrast, an increasing number of studies have examined the benefits of partial-body-coverage compression garments (thigh length or knee length; Ali et al., 2010, 2011; Barwood et al., 2013; Rugg and Sternlicht, 2013; Del Coso et al., 2014; Stickford et al., 2015), and no consensus on the efficacy of the garments on performance is available. Therefore, it is important to determine the optimal compressed area preventing a performance decrease during prolonged running.

To date, only one study has directly evaluated the influence of the body coverage area of compression garments during running (Sperlich et al., 2010). In that study, the subjects performed 15 min of running at 70% of $\dot{\text{V}}$O_2_max followed by an incremental running test until exhaustion while wearing one of three garments (thigh length, knee length, or whole body). However, physiological responses (e.g., oxygen uptake, lactate concentration, and oxygen saturation) and performance parameters (time to exhaustion) were not influenced by the body coverage area. The lack of differences among garments may be explained by the relatively short exercise duration, because performance decrement of lower-body muscles is particularly accelerated during the latter half of prolonged (>60 min) running (Del Coso et al., 2013). Furthermore, prolonged running elevates indirect markers of muscle damage and inflammatory cytokines in the blood, which are associated with impaired muscle strength following exercise (Nieman et al., 2001; Del Coso et al., 2012). However, the effects of partial-body-coverage compression garments on exercise performances [running economy (oxygen uptake), muscle strength], muscle damage, and the inflammatory response during prolonged (>60 min) running are not fully understood (Born et al., 2013). Therefore, the present study examined the effects of the body coverage area of compression garments on the exercise performances and muscle damage during prolonged running (120 min).

## Methods

### Subjects and group classification

Thirty male subjects participated in the present study. Subjects were randomly assigned to one of three groups: (1) wearing compression tights (covering hip to knee) exerting ~15 mmHg at the thigh [CT group; *n* = 10; mean ± standard error (SE) age: 21.3 ± 0.4 years; height: 173 ± 1.4 cm; weight: 65.5 ± 1.5 kg; body mass index: (BMI) 22.2 ± 0.5 kg/m^2^; $\dot{\text{V}}$O_2_max: 53.5 ± 1.2 ml/kg/min]; (2) wearing compression socks (covering knee to ankle) exerting ~15 mmHg at the calf (CS group; *n* = 10; mean ± SE age: 21.6 ± 0.8 years; height: 175.2 ± 1.4 cm; weight: 67.8 ± 2.1 kg; BMI: 22.3 ± 0.7 kg/m^2^; $\dot{\text{V}}$O_2_max: 54.7 ± 1.3 ml/kg/min); and (3) wearing a lower-body garment (covering hip to ankle) exerting no specific pressure level (<5 mmHg) at the thigh or calf (CON group; *n* = 10; mean ± SE age: 22.9 ± 0.7 years; height: 173.4 ± 2.2 cm; weight: 69.2 ± 2.5 kg; BMI: 22.9 ± 0.5 kg/m^2^; $\dot{\text{V}}$O_2_max: 54.0 ± 1.5 ml/kg/min). All subjects were physically active (exercising at least 1 day per week) and had several years of experience in performing sports. Exclusion criteria included a history of inflammatory conditions, musculoskeletal injuries, or chronic pain. Participants were instructed not to consume caffeine or alcohol for at least 24 h prior to the experiment and to refrain from strenuous activity for at least 72 h prior to testing. All subjects gave written informed consent after being informed of the purpose and risks associated with the experiment. This study was approved by the Ethics Committee of Ritsumeikan University, Japan.

### Experimental procedure

All subjects visited the laboratory three times from $\dot{\text{V}}$O_2_max test to main experiment. On the first day, they completed an incremental running test on a treadmill (Valiant, Lode B.V., Groningen, Netherlands) to determine $\dot{\text{V}}$O_2_max. The initial velocity was set at 4 km/h for 3 min, and the velocity was increased by 2 km/h every 3 min. All subjects were required to walk during both the 4 km/h and 6 km/h stages and then began running from the 8 km/h stage. After completing the above three stages of submaximal exercise (9 min after exercise onset), running velocity was increased by 0.6 km/h every minute until exhaustion. The gradient of the treadmill was set at 7% throughout the test. The exercise protocol was designed based on our previous study aimed to determine the influence of the pressure level of compression garments during 120 min of running (Mizuno et al., 2017). Respiratory gases were collected and analyzed using an automatic gas analyzer (AE300S; Minato Medical Science, Tokyo, Japan). Subjects also sufficiently practiced the appropriate measurement procedures for determinations of the maximum voluntary contraction (MVC) of knee extension and plantar flexion, jump performances [counter movement jump (CMJ) and drop jump (DJ)] and subjective feelings of muscle soreness and fatigue. For MVC and jump performance, practices were continued until the peak torque and jump height reached steady state.

On the second day, the pressure level applied while wearing the compression garments was evaluated individually for each garment using an air-packed sensor (AMI3037-2; AMI Techno, Tokyo, Japan). Several types of garments were prepared (different widths) for each group (five garments for the CT and CS groups and three garments for the CON group). An appropriate garment was selected to ensure an equal level of pressure among all participants. The sensor was placed between the skin and garment at the thigh (50% distal point between the greater trochanter and patellar tendon) and/or the calf (30% distal point between the patellar tendon and lateral malleolus) while wearing the prescribed garment. Subjects were instructed to maintain a standing position for 15 s while recording the pressure level, and the mean value was calculated. The compression levels at the thigh and calf were manipulated to match ~15 mmHg for the CT and CS groups and <5 mmHg for the CON group. The compression level of each garment is presented in Table 1. Each garment was custom-made by a sportswear manufacturer (DESCENTE Ltd., Osaka, Japan). Because different levels of pressure among subjects may mask the efficacy of the garment (MacRae et al., 2011), we used custom-made garments to match the pressure level among subjects. Furthermore, based on our previous findings (Mizuno et al., 2017), the applied pressure of the garment was unified to ~15 mmHg at the calf and thigh.

**Table 1:** Compression levels exerted by each garment.

	**Compression at thigh (mmHg)**	**Compression at calf (mmHg)**
CT	14.7 ± 0.6	–
	[12.9–18.3]	
CS	–	17.4 ± 0.5
		[15.2–19.0]
CON	3.0 ± 0.3^†^	1.8 ± 0.2^†^
	[1.4–4.5]	[1.0–3.2]

Values are means ± SE.

† *P < 0.05 vs. CT or CS; CT, compression garment on thigh; CS, compression garment on the calf; CON, lower-body garment on the thigh and calf. The values in parentheses indicate minimal and maximal pressure levels among all subjects*.

On the third visit, subjects arrived at the laboratory following an overnight fast and performed 120 min of uphill running (gradient: 7%) on the treadmill (Elevation series E95Ta; Life Fitness Corporation, Japan) at 55% of $\dot{\text{V}}$O_2_max. Running velocity (5.9 ± 0.1 km/h) was maintained during running in all subjects. Subjects wore the prescribed garments throughout the 120 min of uphill running and rested for 180 min after running while wearing a normal garment without a specific pressure. Changes in HR, rating of perceived exertion (RPE) for the lower leg and respiration, and running economy were monitored. Before exercise and 60 and 180 min after exercise, the MVC of knee extension and plantar flexion, jump performances (CMJ and DJ), thigh and calf circumferences, and subjective feelings (muscle soreness and fatigue) were evaluated to determine the changes in each parameter over time. Blood samples were also collected five times: before exercise (after 20 min of rest), at 60 min of exercise, immediately after exercise, and 60 min and 180 min after exercise. The room temperature during running was kept consistently at 24°C, with a relative humidity of 50% for all trials.

### HR and RPE

HR and RPE for the lower leg and respiration were monitored every 10 min during uphill running (Mizuno et al., 2017). HR was measured continuously using a wireless HR monitor (RCX5; Polar, Tokyo, Japan). The RPE for the lower leg and respiration was recorded using a modified Borg scale from 0 (nothing at all) to 10 (maximal exertion; Borg, 1982).

### Running economy

Expired gas was collected during running to assess running economy using a breath-by-breath method with an automatic analysis system (AE300S, Minato Medical Science Co., Ltd., Osaka, Japan). Data were collected for 5 min during running (at 25–30 min, 55–60 min, 85–90 min, and 115–120 min), and these values were averaged every 30 s. The mean value obtained from the last minute of the respective time point was used for further analysis.

### Jump performance

CMJ and DJ were performed to evaluate changes in power output for the lower limb muscles. All subjects performed CMJ on a jump mat (multi jump tester; DKH Corp., Tokyo, Japan) connected to a computer. Subjects were instructed to jump as high as possible while placing their hands on the lumbar division to eliminate any arm-swing effect. The flight and contact time were recorded during the vertical jump. The CMJ height was calculated from the flight time using the following formula (Mizuno et al., 2017);

$$\begin{array}{lll} Jump\;height\;\left(cm\right)\; & = & \;\frac{1}{8}\;{\left(flight\;time\right)}^{2}\;\times \;gravity\;constant\\ \left(=\;9.81\;m/s^{2}\right). \end{array}$$

Subjects performed DJ from a 60-cm box. After landing on a platform, the subject was instructed to perform a maximal vertical jump with minimal contact time. The DJ index was calculated from the jump height and contact time (jump height/contact time).

Each jump test was repeated twice with 2 min rest period between jumps, and the highest CMJ height and DJ index were used for analysis. The intraclass correlation coefficients for CMJ height and DJ index were 0.97 and 0.81, respectively.

### MVC of knee extension and plantar flexion

The MVC (right leg) of knee extension and plantar flexion was assessed using an isokinetic dynamometer (Biodex System 4, SAKAI Medical Co., Ltd., Tokyo, Japan). Subjects were seated on a chair, and straps were used to fix the chest, hip, waist, and thigh during the measurements. For knee extension, the right ankle was firmly attached to the lever of the dynamometer by a strap, and the pivot of the lever was set at the knee joint. The MVC was evaluated at a knee angle of 75° (full extension of the lower leg was expressed as 0°). For plantar flexion, the right foot was placed on a footplate with a fixed instep. The pivot of the lever was adjusted for the lateral malleolus. The MVC of plantar flexion was measured at a 0° dorsiflexion position with full extension of the lower leg. Alignment of the joint angles and dynamometer axes were maintained during MVC of knee extension and plantar flexion. Two 3-s contractions were performed with 1 min of rest between contractions, and the peak torque value was used for further analysis (Goto and Morishima, 2014). Verbal encouragement was provided during tests. The intraclass correlation coefficients for the MVC of knee extension and plantar flexion were 0.92 and 0.97, respectively.

### Scores of subjective muscle soreness and fatigue

Subjective muscle soreness and fatigue were assessed using a 10-cm visual analog scale, with 0 cm indicating no pain or fatigue, and 10 cm indicating the worst pain and fatigue. After two knee-bend actions, muscle soreness was evaluated at three sites: the anterior and posterior thigh and calf.

### Thigh and calf circumferences

To evaluate muscle swelling resulting from exercise, the thigh (50% distal point between the greater trochanter and patellar tendon) and calf (30% distal point between the patellar tendon and lateral malleolus) circumferences were measured in anatomical positions using a tape measure. The sites used for measurement were marked before exercise, and identical sites were used throughout the study to ensure precision of the measurements.

### Blood sampling and analysis

Blood samples from the antecubital vein were collected to determine blood glucose, lactate, serum myoglobin (Mb), free fatty acid (FFA), high-sensitivity C reactive protein (hsCRP), and plasma IL-6 concentrations. Serum and plasma samples were obtained by centrifugation (3,000 rpm, 10 min, 4°C). Samples were stored at −60°C until analysis. Blood glucose and lactate concentrations were measured using an automatic glucose analyzer (Freestyle, Nipro Corp., Osaka, Japan) and a lactate analyzer (Lactate Pro, Arkray Inc., Kyoto, Japan), respectively. Serum Mb, FFA, and hsCRP concentrations were measured at the SRL Clinical Laboratory in Tokyo, Japan. Plasma IL-6 concentrations were assayed using an enzyme-linked immunosorbent assay (ELISA) kit (R&D Systems, Minneapolis, MN, USA). The intra-assay coefficients of variation for each measurement were as follows: 2.2% for Mb, 1.1% for FFA, 2.4% for hsCRP, and 4.7% for IL-6.

### Statistical analysis

Data are expressed as the mean ± SE. Changes in physiological (e.g., MVC, jump performance, thigh/calf circumference, running economy, and blood variables) and psychological (e.g., RPE and subjective feelings) variables were initially analyzed using two-way analysis of variance (ANOVA) with repeated measures (group and time). When ANOVA revealed a significant interaction or main effect, the Tukey-Kramer *post*-*hoc* test was used to assess the differences. For the comparisons of mean HR and RPE, and exerted prssure levels, one-way ANOVA with a *post*-*hoc* test was performed. The significance level was set at *P* < 0.05.

## Results

### Compression level of each garment

Table 1 presents the compression level exerted by each garment. The compressive pressure at the thigh or calf was significantly lower in the CON group than in the CT and CS groups (*P* < 0.001).

### HR, RPE, and running economy

**Figure 1:**
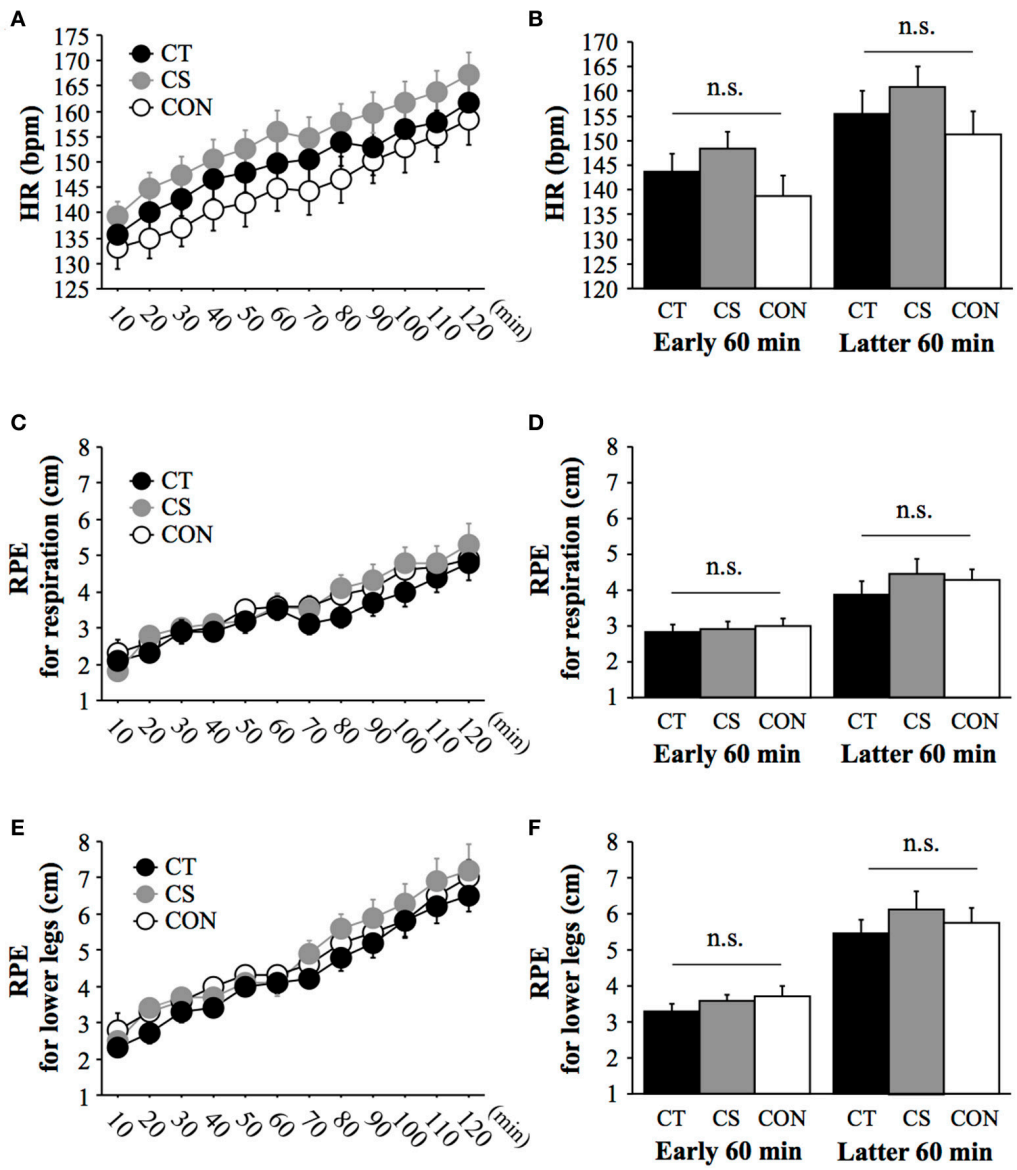
Heart rate (HR) **(A)**, mean HR values **(B)**, rating of perceived exertion (RPE) for respiration **(C)**, mean RPE values for respiration **(D)**, RPE for legs **(E)** and mean RPE values for legs **(F)** during 120 min of running. Values are mean ± standard error. n.s., no significant difference among groups.

The changes in HR and RPE throughout the exercise are presented in Figure 1. All variables increased gradually with exercise (main effect for time: *P* < 0.001). However, the responses were similar among the three groups (group × time: *P* > 0.05; main effect for group: *P* > 0.05).

The $\dot{\text{V}}$O_2_ during exercise (running economy) did not show a significant interaction (group × time: *P* = 0.54) or main effect for group (*P* = 0.25). However, a significant main effect for time was observed (*P* < 0.001).

### Jump performance and MVC

**Figure 2:**
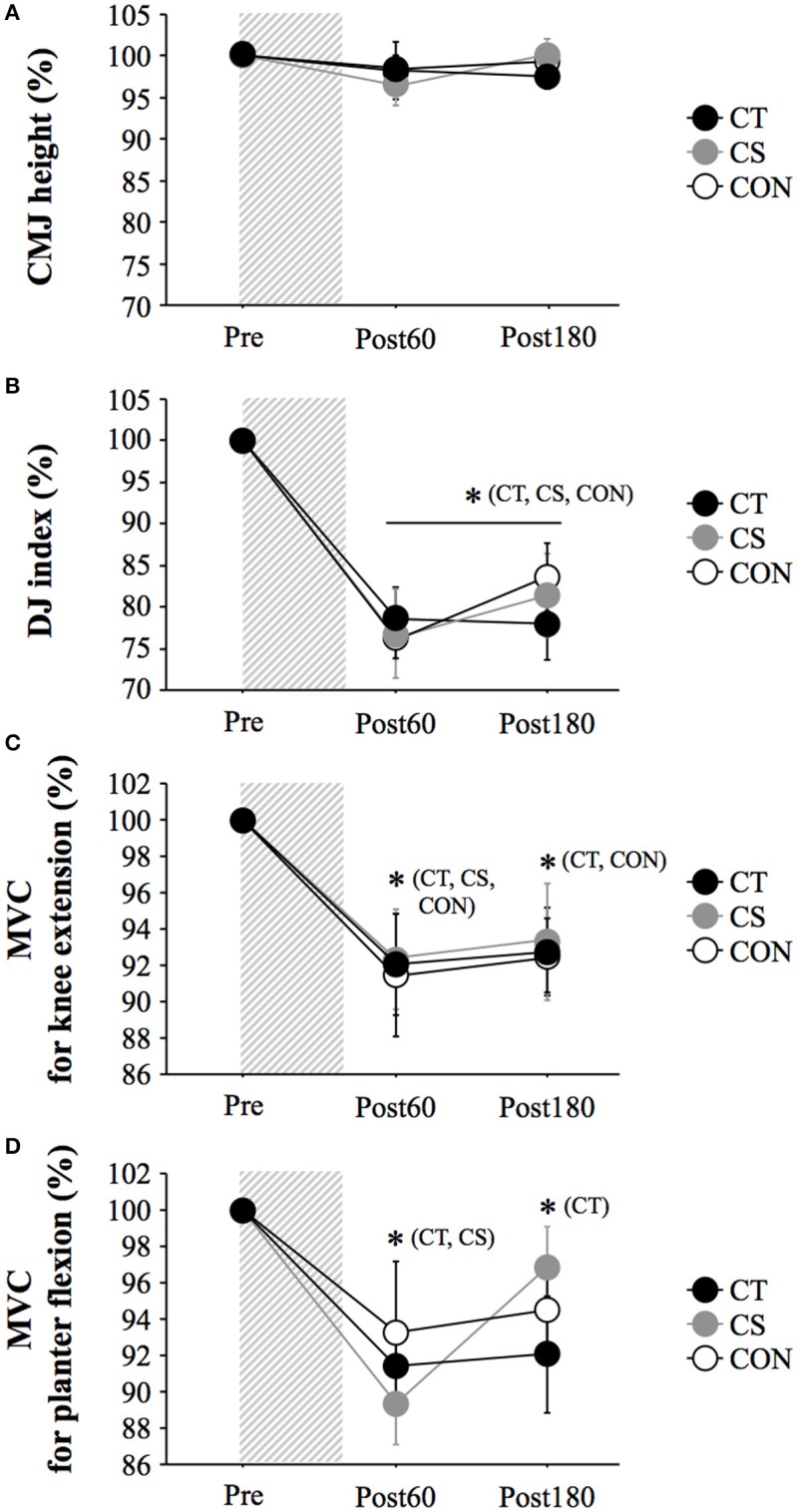
Percent change in counter-movement jump (CMJ) height **(A)**, drop jump (DJ) index **(B)**, maximal voluntary contraction (MVC) for knee extension **(C)** and MVC for planter flexion **(D)**. Values are mean ± standard error. The gray bar indicates a duration of 120 min uphill running. ^*^P < 0.05 vs. Pre. Post60, Post180, 60 min, and 180 min after exercise.

Figure 2 shows the changes in MVC and jump performances. No significant difference among 3 groups was detected for any parameter at baseline (Pre). The MVC of knee extension and plantar flexion was significantly reduced after exercise (main effect for time: *P* < 0.001). However, there was no significant difference among the three groups at any time (group × time: *P* > 0.05; main effect for group: *P* > 0.05). No significant interaction (group × time: *P* = 0.70) or main effect for group (*P* = 0.93) or time (*P* = 0.18) was observed for CMJ height. Although all groups showed a significant decrease in the DJ index during the post-exercise period (main effect for time: *P* < 0.001), there was no significant interaction (group × time: *P* = 0.70) or main effect for group (*P* = 0.98).

### Scores for subjective muscle soreness and fatigue

Scores for subjective muscle soreness and fatigue were significantly elevated during the post-exercise period (main effect for time: *P* < 0.001). However, no significant interaction (group × time) or main effect for group was detected.

### Thigh and calf circumferences

No significant interaction (group × time) or main effect for group was observed for thigh or calf circumference. All groups showed a significant reduction in thigh and calf circumferences during the post-exercise period (main effect for time: *P* < 0.001).

### Blood variables

Table 2 presents the changes in blood glucose, lactate, serum FFA, hsCRP, and plasma IL-6 concentrations. There was no significant difference in any of the blood variables among the three groups at baseline (Pre). The blood glucose concentration was significantly decreased during the post-exercise period (main effect for time: *P* < 0.001). However, there was no significant difference among the three groups (group × time: *P* = 0.70, main effect for group: *P* = 0.65). The blood lactate concentration revealed a significant main effect for time (*P* = 0.03), but no significant interaction (group × time: *P* = 0.66) or main effect for group (*P* = 0.88). The serum FFA concentration increased markedly with time in all groups (main effect for time: *P* < 0.001); however, there was no significant interaction (group × time: *P* = 0.82) or main effect for group (*P* = 0.10). The serum hsCRP concentration did not reveal a significant interaction (group × time: *P* = 0.12) or main effect for group (*P* = 0.15). However, the serum hsCRP concentration increased significantly during the post-exercise period in the CT group (main effect for time: *P* < 0.001), but not in the CS or CON group. A significant increase in plasma IL-6 concentration over time was observed (main effect for time: *P* < 0.001), with similar responses among the three groups (group × time: *P* = 0.59; main effect for group: *P* = 0.19).

**Table 2:** Time course changes in blood glucose, lactate and serum free fatty acid (FFA) and high sensitive C-reactive protein (hsCRP), and plasma interleukin-6 (IL-6) concentrations.

		**Pre**	**Ex60**	**Ex120**	**Post60**	**Post180**
Glucose (mg/dL)	CT	83 ± 1	84 ± 2	78 ± 4	73 ± 2^*^	74 ± 2
	CS	85 ± 4	82 ± 3	79 ± 3	73 ± 3^*^	72 ± 3^*^
	CON	89 ± 4	84 ± 3	72 ± 3^*^	72 ± 3^*^	76 ± 3^*^
Lactate (mmol/L)	CT	1.5 ± 0.1	1.3 ± 0.2	1.3 ± 0.1	1.6 ± 0.1	1.5 ± 0.1
	CS	1.4 ± 0.1	1.2 ± 0.2	1.8 ± 0.4	1.7 ± 0.2	1.5 ± 0.1
	CON	1.3 ± 0.3	1.1 ± 0.1	1.5 ± 0.1	1.6 ± 0.2	1.5 ± 0.1
FFA (mmol/L)	CT	0.44 ± 0.06	0.83 ± 0.08^*^	1.67 ± 0.09^*^	1.17 ± 0.07^*^	1.09 ± 0.13^*^
	CS	0.57 ± 0.09	0.95 ± 0.11^*^	1.94 ± 0.15^*^	1.34 ± 0.09^*^	1.19 ± 0.08^*^
	CON	0.39 ± 0.05	0.81 ± 0.15^*^	1.54 ± 0.16^*^	1.33 ± 0.09^*^	0.97 ± 0.08^*^
hsCRP (μg/dL)	CT	118 ± 19	125 ± 20	127 ± 20	128 ± 18^*^	133 ± 19^*^
	CS	427 ± 71	258 ± 67	267 ± 70	250 ± 69	284 ± 75
	CON	322 ± 49	204 ± 47	203 ± 46	202 ± 46	208 ± 44
IL-6 (pg/mL)	CT	0.7 ± 0.1	1.4 ± 0.2	4.0 ± 0.9^*^	2.6 ± 0.4^*^	2.5 ± 0.8
	CS	0.6 ± 0.8	1.8 ± 0.4	5.7 ± 1.2^*^	3.5 ± 0.7^*^	2.1 ± 0.5
	CON	0.7 ± 0.1	1.4 ± 0.3	4.0 ± 0.7^*^	2.7 ± 0.3^*^	1.5 ± 0.2

Values are means ± SE. CT, compression garment on thigh; CS, compression garment on the calf; CON, lower-body garment on the thigh and calf.

* *P < 0.05 vs. Pre. Ex60, 60 min of exercise; Ex120, immediately after exercise. Post60, Post180, 60 min and 180 min after exercise*.

**Figure 3:**
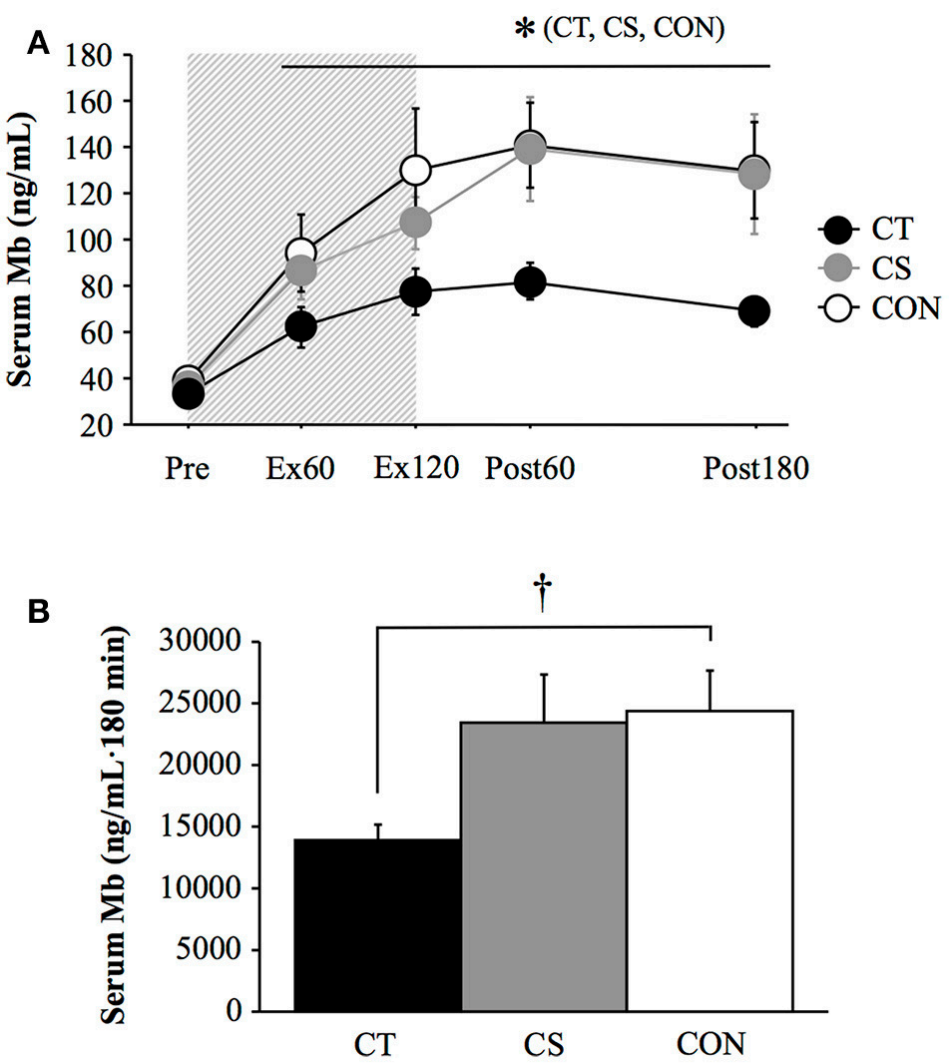
Serum myoglobin (Mb) concentrations **(A)** and area under the curve for Mb during 180 min of post-exercise period **(B)**. Values are mean ± standard error. The gray bar indicates a duration of 120 min uphill running. ^*^P < 0.05 vs. Pre. ^†^*P* < 0.05 between CT and CON. CT, compression garment on the thigh; CON, lower-body garment on the thigh and calf; Ex60, 60 min during exercise; Ex120, immediately after exercise. Post60, Post180, 60 min and 180 min after exercise.

For the serum Mb concentration, a significant interaction (group × time: *P* = 0.03) and main effect for time (*P* < 0.001) were observed. Furthermore, the area under the curve during the post-exercise period (180 min) was significantly lower in the CT group (13,833 ± 1,397 ng/mL) than in the CON group (24,343 ± 3,370 ng/mL, *P* = 0.03; Figure 3). No significant difference was observed between the CS and CON groups.

## Discussion

To date, various types of compression garments (e.g., tights, socks, stockings, and whole-lower-body garments) have been utilized during exercise. However, it is unknown whether the body coverage area affects the benefits of the garment. Therefore, the purpose of the present study was to investigate the effects of wearing compression garments with different body coverage areas on exercise performances and muscle damage during 120 min of uphill running. The present findings revealed no significant effects of the body coverage area of the compression garments on the selected exercise performances (MVC and jump performances), running economy ($\dot{\text{V}}$O_2_), or scores of subjective feelings. However, the increase in Mb concentration induced by prolonged running was significantly attenuated in the CT group compared with the CON group, suggesting that exercise-induced muscle damage was attenuated by a wearing a compression garment covering the thigh.

Exercise-induced muscle damage is characterized by increases in circulating concentrations of intramuscular enzymes, such as, creatine kinase and Mb, resulting from increased cellular permeability and/or histological damage (Chen et al., 2011). Interestingly, the exercise-induced increase in Mb concentration was significantly lower in the CT group compared with the CON group. Borràs et al. (2011) also reported that wearing compression tights during running significantly attenuated histological muscle damage of the vastus lateralis following running. Furthermore, muscle oscillation during running was concomitantly decreased when the thigh was covered by a compression garment. Because the decrease in muscle oscillation could reduce mechanical stress in tissues (Valle et al., 2013), the attenuated elevation of the Mb concentration in the CT group may be associated with a reduction in muscle oscillation in the thigh area (Kraemer et al., 1998; Doan et al., 2003), However, we did not observe a significant difference in the MVC of knee extension among the groups. Furthermore, since the phenomenon of exercise-induced muscle damage lasts for several days following a damage-inducing exercise, future research should clarify whether attenuated Mb elevation prevents the increases in muscle damage markers after 180 min of post-exercise.

The use of compression garments is believed to improve endurance capacity (Bringard et al., 2005) by reducing the muscle oscillations of working muscles, which could subsequently lessen exercise-induced muscle damage (MacRae et al., 2011). Furthermore, application of compression garments may enhance the removal of metabolites (e.g., inorganic phosphate and H^+^) from muscles associated with augmented peripheral circulation, leading to reduced muscle fatigue (Valle et al., 2013; Miyamoto and Kawakami, 2014). However, we did not detect a significant difference in MVC or jump performances among the three groups. The present findings are consistent with previous results in which wearing compression tights or socks during running did not prevent the exercise-induced decrease in maximal muscle strength (Vercruyssen et al., 2014, 2017) or jump performance (Higgins et al., 2009; Ali et al., 2010; Del Coso et al., 2014). Taken together, wearing a partial-body (e.g., thigh or calf)-coverage garment may not benefit muscle function or reduce muscle fatigue during exercise. Furthermore, running economy during exercise did not differ significantly among the groups in the present study. Bringard et al. (2005) demonstrated that application of compression tights improved economy of oxygen uptake (decreased slow component of $\dot{\text{V}}$O_2_ elevation) during submaximal running, although the finding was not consistent with other studies (Ali et al., 2010; Sperlich et al., 2010, 2011; Stickford et al., 2015). Born et al. (2013) reported that the lack of an effect on physiological variables may be due to insufficient or inappropriate pressure intensities applied by compression garments. In the current study, the pressure level was carefully matched at 15 mmHg based on our previous study using whole-lower-body garments during 120 min of uphill running (Mizuno et al., 2017). These findings suggest that the optimal pressure intensity required to obtain physiological advantages may depend on the body coverage area of the compression garment. In addition, the attenuated increase in Mb concentration in the CT group was not accompanied by an improvement in exercise performance. The lack of an effect could be due to different time course changes between muscle strength and serum muscle enzyme levels, which do not necessarily correlate with each other (Suzuki et al., 2006).

No significant differences in subjective feelings of muscle soreness or fatigue were observed among the groups. Increased muscle soreness following exercise reflects tissue disruption and cellular damage, which are associated with an inflammatory response and subsequent muscle swelling (Paulsen et al., 2012). Correspondingly, we failed to observe any significant difference in inflammatory cytokine (i.e., IL-6) levels or thigh/calf circumferences among the groups. However, these variables were monitored during the relatively initial phase (180 min) of the post-exercise period, and thus we are unable to draw conclusions regarding the secondary muscle damage response generally observed during several days following damaging exercise.

Wearing a compression garment during exercise is believed to increase venous return, with an increased stroke volume and concomitant reduction in HR (MacRae et al., 2011). Considering that muscle pump action is dependent on exercise intensity, the impact of wearing a compression garment would be profound under a lower running velocity (lower level of venous return). This notion is supported by a previous finding demonstrating that the exercise-induced HR elevation was significantly less when wearing a lower-body compression garment while running under 6 km/h, but not at 10 km/h and 80% of $\dot{\text{V}}$O_2_max (Lovell et al., 2011). However, we did not observe a decrease in the exercise-induced HR elevation during running, despite the lower running velocity (5.9 ± 0.1 km). These inconsistent results may be attributed to differences in the body coverage area of the compression garments. The attenuated HR elevation was only identified when wearing a whole-lower-body compression garment (Dascombe et al., 2011; Lovell et al., 2011; Mizuno et al., 2017), while partial-body-coverage compression garments (e.g., tights, stockings, and socks) failed to show favorable changes in HR during exercise (Bringard et al., 2005; Rimaud et al., 2010; Ali et al., 2011). These findings suggest that compressing both the thigh and calf may be indispensable for attenuating the exercise-induced HR elevation. Further research is warranted to determine whether different body coverage areas of compression garments decrease cardiovascular strain during prolonged exercise.

## Conclusion

Wearing compression tights exerting ~15 mmHg to the thigh significantly attenuated the exercise-induced elevation of serum Mb during 120 min of running. However, exercise-induced changes in maximal muscular strength, jump performances, subjective feelings, and thigh/calf circumferences did not differ significantly regardless of different coverage area of the compression garment.

## Author contributions

SM and KG conceptualized and designed the study and contributed to analysis and interpretation. SM performed the data collection and drafting of the paper. KG revised the work and final approval of the manuscript. MA, FT, and EY contributed to making garments and evaluating pressure levels.

### Conflict of interest statement

MA, FT, and EY were employed by DESCENTE Ltd. The other authors declare that the research was conducted in the absence of any commercial or financial relationships that could be construed as a potential conflict of interest. The authors declare that this study received funding from DESCENTE Ltd. The funder had the following involvement with the study: design, analysis and interpretation.
